# 23 Chlorhexidine Delays Wound Healing in Human Skin

**DOI:** 10.1093/jbcr/irac012.026

**Published:** 2022-03-23

**Authors:** Angela Gibson, Aiping Liu, Collin L Tran, Sameeha E Hassan

**Affiliations:** University of Wisconsin School of Medicine and Public Health, Madison, Wisconsin; University of Wisconsin-Madison, Madison, Wisconsin; University of Wisconsin-Madison, Madison, Wisconsin; University of Wisconsin School of Medicine and Public Health, Madison, Wisconsin

## Abstract

**Introduction:**

Chlorhexidine (CHG) is ubiquitous in surgical perioperative care. *In vivo* studies of CHG cytotoxicity on human skin are lacking. Given the use of CHG for daily wound cares and as a presurgical scrub, including donor site preparation, we sought to identify if CHG cytotoxicity would persist in a clinically relevant *in vivo* human skin xenograft model.

**Methods:**

Human skin tissues were obtained from elective surgeries. Partial thickness wounds were created *ex vivo* in human skin using a 4 mm punch biopsy. 2% CHG (treatment) or PBS (control) was applied to the wounds for 30 minutes followed by rinsing the tissue +/- mechanical disruptive irrigation. Tissues were cultured at the air-liquid interface for 24 hours in culture media after treatment and tissue viability was performed using an MTT assay. For *in vivo* studies, athymic mice (n=4) were grafted on bilateral flanks with human skin. Eight weeks after engraftment and normalization of skin architecture, 4 mm partial thickness wounds were created on each xenograft (2 per mouse – treatment and control). 2% CHG was applied daily for 2 minutes followed by irrigation with PBS in the treatment wound. The control wound received PBS application and irrigation. The xenografts received treatment daily for 14 days to mimic daily wound cares, and digital images were obtained to document presence of infection and gross wound healing. On day 14, the xenografts were harvested and stained for lactate dehydrogenase and H&E to assess cell viability and wound re-epithelization, respectively.

**Results:**

An MTT assay on *ex vivo* human skin wounds showed that CHG treated groups (irrigation or non-irrigation) had lower cell viability compared to the PBS treated group, however irrigation mitigates the cytotoxicity of CHG on human skin. In the *in vivo* xenograft study, no signs of infection were identified in either PBS or CHG treated wounds throughout the study. The wound size appeared larger on gross inspection in the CHG treated group compared to the PBS group as early as day 2.Microscopically, the PBS treated wounds were fully re-epithelialized (n=2) or had significantly more re-epithelialization (n=2) than the CHG treated wounds (n=4) after 14 days of treatment. The PBS-treated wounds were viable throughout the tissue, indicating the irrigation procedure was not harmful to the cells. In the CHG-treated wounds, nonviable cells were observed in the dermis beneath the wound that was directly in contact with CHG suggesting penetration of CHG contributes to cytotoxicity in acute wounds.

**Conclusions:**

Daily CHG use is cytotoxic to human skin and impedes wound healing.

